# Tetracyclic and Pentacyclic Triterpenes with High Therapeutic Efficiency in Wound Healing Approaches

**DOI:** 10.3390/molecules25235557

**Published:** 2020-11-26

**Authors:** Roxana Ghiulai, Oana Janina Roşca, Diana Simona Antal, Marius Mioc, Alexandra Mioc, Roxana Racoviceanu, Ioana Macaşoi, Tudor Olariu, Cristina Dehelean, Octavian Marius Creţu, Mirela Voicu, Codruţa Şoica

**Affiliations:** 1 Department of Pharmaceutical Chemistry, Faculty of Pharmacy, Victor Babeş University of Medicine and Pharmacy, 2nd Eftimie Murgu Sq., 300041 Timişoara, Romania; roxana.ghiulai@umft.ro (R.G.); janinarosca@yahoo.com (O.J.R.); marius.mioc@umft.ro (M.M.); babuta.roxana@umft.ro (R.R.); codrutasoica@umft.ro (C.Ş.); 2 Department of Pharmaceutical Botany, Faculty of Pharmacy, Victor Babeş University of Medicine and Pharmacy, 2nd Eftimie Murgu Sq., 300041 Timişoara, Romania; 3 Department of Anatomy, Physiology, Pathophysiology, Faculty of Pharmacy, Victor Babeş University of Medicine and Pharmacy, 2nd Eftimie Murgu Sq., 300041 Timişoara, Romania; alexandra.petrus@umft.ro; 4 Department of Toxicology, Faculty of Pharmacy, Victor Babeş University of Medicine and Pharmacy, 2nd EftimieMurgu Sq., 300041 Timişoara, Romania; macasoi.ioana@umft.ro (I.M.); cadehelean@umft.ro (C.D.); 5 Department of Organic Chemistry, Faculty of Pharmacy, Victor Babeş University of Medicine and Pharmacy, 2nd EftimieMurgu Sq., 300041 Timişoara, Romania; olariu.t@umft.ro; 6 Department of Surgery, Faculty of Medicine, Victor Babeş University of Medicine and Pharmacy, 2nd EftimieMurgu Sq., 300041 Timişoara, Romania; tavicretu@yahoo.com; 7 Department of Pharmacology, Faculty of Pharmacy, Victor Babeş University of Medicine and Pharmacy, 2nd EftimieMurgu Sq., 300041 Timişoara, Romania

**Keywords:** wound healing, pentacyclic triterpenes, tetracyclic triterpenes

## Abstract

Wounds are among the most common skin conditions, displaying a large etiological diversity and being characterized by different degrees of severity. Wound healing is a complex process that involves multiple steps such as inflammation, proliferation and maturation and ends with scar formation. Since ancient times, a widely used option for treating skin wounds are plant- based treatments which currently have become the subject of modern pharmaceutical formulations. Triterpenes with tetracyclic and pentacyclic structure are extensively studied for their implication in wound healing as well as to determine their molecular mechanisms of action. The current review aims to summarize the main results of in vitro, in vivo and clinical studies conducted on lupane, ursane, oleanane, dammarane, lanostane and cycloartane type triterpenes as potential wound healing treatments.

## 1. Introduction

The skin is an effective barrier against all external factors including microorganisms, also providing other important physiological functions such as thermoregulation, fluid regulation, sensory detection and even vitamin synthesis [1]. A wound represents a discontinuity of the epitelial integrity that needs rapid restoration in order for the skin to regain its vital functions [2]. Wounds can be classified by: (1) their onset, as acute or chronic, or (2) their etiology, as surgical incisions which provide minimal tissular destruction, and traumatic wounds such as abrasions, lacerations, contusions, open wounds as well as, thermal, physical and chemical burn wounds [3]. Wounds can be limited to superficial skin layers or go deeper and affect the blood vessels, nerves, muscles, ligaments, tendons and even bones in certain severe traumatic conditions. The skin has the ability to heal itself through a complex process but often local or systemic factors such as infection, alterations in blood supply of venous drainage, foreign body as well as diabetes, cancer, chemotherapy, vasculitis, obesity, anticoagulant treatment or immunosuppressive therapy interfere and delay wound healing [4].

Consistent with WHO recommendations, the management of open wounds must eliminate any possibility of infection, because it may lead to chronic lesions of more severe outcomes, such as bone infection, necrosis or even death; thus in addition to all the procedures of wound cleaning, a course of systemic antibiotics such as penicillin G and/or metronidazole may be indicated [5]. Also, very often, various wound dressings are applied as standard procedure to promote wound healing, either passive dressings or active/interactive dressings such as alginate, hydrogels, hydrocolloids, semipermeable films, foams or antimicrobial dressings [6]. Furthermore, among the strategies involved in the management of wound healing, bioengineered skin is currently being used, either as autografts or allografts (epidermal, dermal or composite grafts), accompanied by some non-surgical procedures such as vacuum-assisted closure, intermittent pneumatic compression or hyperbaric oxygen; also, several drugs that produce vasodilation or blood thinning such as pentoxifylline, iloprost, calcium channel blockers and nitroglycerin can be recommended [7]. Future perspectives include the use of epidermal stem cells, previously demonstrated in preclinical studies as highly efficient for wound repair and currently undergoing several clinical trials with confirmed results; thus, tissue-engineered epidermal stem cells are considered a potential treatment for wound healing and repair [8]. Last but not least, ever since ancient times, medicinal plants and plant-based pharmaceutical formulations, rich in various types of bioactive phytocompounds, are important and widely used therapeutic options in the management of wound healing due to either their implication in accelerating the wound healing process or their antimicrobial activity and infection prevention [9]. Among the most popular plants, *Aloe vera, Calendula officinalis, Camellia sinensis, Centella asiatica, Curcuma longa* or *Panax ginseng* are widely recognized as efficient and safe remedies for wound treatment [10].

Terpenoids or isoprenoids are a widespread class of plant-derived phytochemicals, secondary metabolites with a large variety of chemical structures, all derived from isoprene, and most of them with polycyclic structure [11]. They are classified based on the number of isoprene units identified in their structures in hemiterpenes, monoterpenes, sesquiterpenes, diterpenes, triterpenes and tetraterpenes; triterpenes possess pentacyclic or tetracyclic structure [12]. Pentacyclic triterpenes are subdivided based on their core structure in lupane, oleanane and ursane derivatives, many of them with proved anti-cancer, chemopreventive, antiinflamatory, antioxidant, cardio and hepato-protective or anti-viral activities [13,14,15], while among tetracyclic triterpenes, dammarane-, lanostane- or cycloartane-type are the most studied for their cytotoxic and anti-cancer biological properties [14,16]. Recently, systematic studies were conducted in order to evaluate the efficacy of triterpenes in the process of wound healing; results indicated that these classes of phytocompounds are able to speed up the healing process by accelerating epithelialization and collagen production and deposition, irrespective of the wound type; moreover, their incorporation in various medicinal formulations is a valuable option in the management of wounds due to their long-term delivery of active phytocompounds. Therefore, the literature reports triterpenic phytocompounds as being considered an emerging class of therapies in the treatment and management of wounds [17].

This review summarizes the biological activities of triterpenic phytocompounds with pentacyclic and tetracyclic structure, such as lupane, ursane, oleanane, dammarane, lanostane and cycloartane derivatives in wound healing, demonstrated in extensive in vitro and in vivo studies by highlighting their molecular mechanisms of action; in addition, data collected from clinical trials conducted on these classes of purified phytocompounds or on complex vegetal extracts were reviewed in order to support their evidence-based therapeutic effect in wound healing. Also, another important aspect is an overview of the main innovative nano-formulations containing triterpenes that were demonstrated to enhance their bioavailability and effectiveness in wound management, thus being promising candidates for a new class of medicinal products of dermatological use.

## 2. Skin Anatomy and Wound Healing

**Figure 1:**
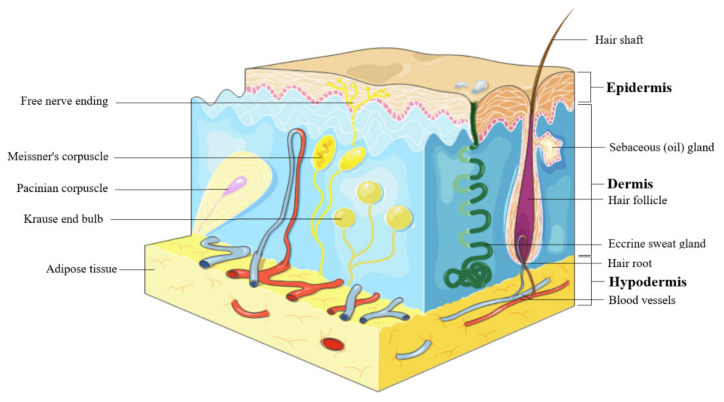
Skin structure.

The skin represents the largest organ of the body and consists in 3 layers: epidermis, dermis, and hypodermis (Figure 1). Epidermis is the outer most layer of the skin and is stratified in 5 layers such as: (1) *stratum basale*, the deepest layer of epidermis that contains melanocytes, (2) *stratum spinosum* or squamous cell layer, (3) *stratum granulosum* that contains keratohyalin granules and keratin precursors, (4) *stratum lucidum*, and (5) *stratum corneum*, the uppermost layer that contains anucleate keratinocytes, also known as corneocytes, mostly made up by keratin, and acts as a barrier against external factors and water loss [18]. The epidermis cells include (i) keratinocytes, able to produce keratin and to act as epidermal water barrier, (ii) melanocytes that secrete melanin, the skin’s pigment which act as protective shield against UV radiation, (iii) Langerhans’ cells, providing the first line defense, and (iv) Merkel’s cells, possessing sensory functions [19]. Dermis consists in 2 layers, the papillary layer, formed by loose connective tissue with small diameter collagen fibres and the reticular layer, formed by more dense connective tissue with large diameter collagen fibers, mostly type I collagen, accompanied by type III collagen in a lesser extent [20]. Fibroblasts are the main components of dermis, responsible for collagen fiber and elastin production, accompanied by proteoglycans and skin appendages such as sweat glands, hair follicles, muscles, sensory neurons, and blood vessels [19]. Hypodermis or the subcutaneous fascia is formed by adipocytes arranged in lobules and is located between the dermis and the muscles; it serves as a shield for the internal organs against trauma or cold and participates in energy production [21].

**Figure 2:**
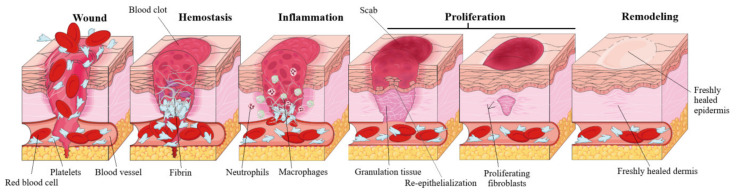
Healing cascade phases of acute wounds.

Acute wound healing, also known as the cascade of healing, is a complex process of four overlapping stages: hemostasis, inflammation, proliferation and remodeling [4] (Figure 2). The first step, hemostasis, consists in the formation of a blood clot in order to stop the bleeding, which eventually dries and forms a solid layer, a scab, that prevents the infection of the underlying tissue. This phase is achieved by rapid vasoconstriction, through (1) the activation of the coagulation cascade during which prothrombin is converted to thrombin which consequently promotes the conversion of fibrinogen to fibrin, and (2) by platelet activation and the consequently formation of a platelet plug [22]. The second stage, the inflammatory phase, is generated by the immune system as a defensive mechanism, resulting in vasodilation that increases the transport of oxygen and nutrients in the wound area and is characterized by the four cardinal signs of inflammation: rubor (erythema), tumor (oedema), dolor (pain), calor (heat) [23]. The inflammatory phase is divided in: (1) the early inflammatory phase, which starts shortly after coagulation and during which the complement cascade is activated, resulting in a neutrophil infiltrate and phagocytosis at the wound site in order to prevent infection, and (2) the late inflammatory phase, 48–72 h after the injury, during which macrofages, attracted by a plethora of chemoattractive agents, take over the fagocytosis; after 72 h lymphocytes arrive at the wound site [24]. During the third stage, proliferation, several major events take place: fibroblast migration, extracellular matrix formation, granulation, contraction and shrinking of the wound followed by reepithelization. Fibroblasts are attracted at the wound site and consequently start to secrete fibronectin, collagen, elastin, hyaluronic acid and proteoglycans, in order to promote extracellular matrix formation, followed by the formation of granulation tissue, a pink and soft tissue that fills the wound; lastly, neoangiogenesis occurs, promoted by vascular endothelial growth factor or platelet derived growth factor [4]. Reepithelization resulting in a red, shiny and tender tissue, is achieved by the migration of basal cells, present at the wound edge, across the wound area, initially as a monolayer which subsequently starts to proliferate [25]. The fourth stage, the maturation or remodeling stage can last up to 2 years, during which the newly formed tissue gains strength and flexibility by reorganization, degradation, and re-synthesis of the extracellular matrix [26]. The result of wound healing varies according to different types of wounds; if the lesion only affects the skin no scar will develop, while for deeper wounds, scars with abnormal aspect, such as keloids, will develop [27].

## 3. Triterpenes with Wound Healing Effects: Mechanism of Action

### 3.1. Lupane-Type Triterpenes

**Figure 3:**
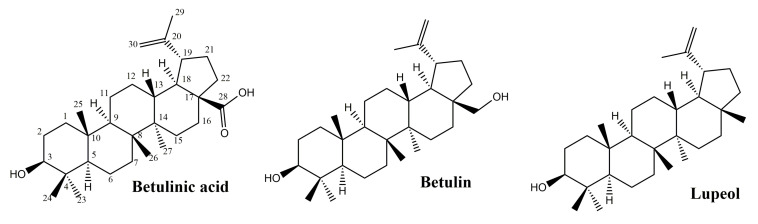
Chemical structures of lupane-skeleton triterpenes.

Pentacyclic triterpenes, secondary metabolites extensively found in the plant kingdom, are located in various parts of the plant, such as the bark, root and leaves. An important number of studies showed the curative potential of lupane-type pentacyclic triterpenes (Figure 3) through various biological activities such as anti-inflammatory [28,29], anticancer [30,31], antioxidant [12], antiviral [13,32], antiparasitic [33] or healing properties [17], all of them being confirmed through extensive research.

#### 3.1.1. Betulinic Acid and Betulin

Betulin, (lup-20(29)-ene-3b,28-diol), is an abundant, natural triterpene found in large amounts in the bark of birch species (*Betula* sp., Betulaceae family). Another naturally occurring pentacyclic triterpenoid is betulinic acid (3b-hydroxy-lup-20(29)-en-28-oic acid) that can be obtained by betulin oxidation and exhibits various pharmacological activities among which the antioxidant and anti-inflammatory effects are of interest in wound healing [34].

*Dillenia indica* fruit extracts standardized in betulinic acid were tested in vitro on egg yolk (lipid peroxidation test) and in vivo on male albino Wistar rats, with ultraviolet induced psoriasis-like wounds [35]. The in vitro studies indicated that the *D. indica* extracts can provide a substantial protection against lipid peroxidation, while a significant speed-up healing process was observed on induced wounds of rat tail, when extracts with 50 mg/mL concentration in betulinic acid were used. As revealed by the histological examination, *D. indica* extracts produced a diminished immune cell infiltration in the granulation tissue and also limited para-keratosis. Moreover, the tested extracts exhibited an important anti-inflammatory effect and presented accelerated wound healing properties on psoriasis-like wounds [35]. In another study, betulinic acid isolated from *Diospyros kaki* was investigated for the anti-inflammatory effects in lipopolysaccharide-stimulated RAW264.7 macrophages [36]. Macrophages play an important role in the inflammatory process, being part of the formation of pro-inflammatory facilitators; the study pointed out that betulinic acid determined HO-1/Nrf2 translocation by suppressing the NF-κB pathway. The suppressing effect on pro-inflammatory cytokines IL-1β, TNF-α and IL-6 as well as the inhibition of COX-2 and iNOS determined by ELISA assays were dose-dependent, confirming yet again the anti-inflammatory effect of betulinic acid [36]. In perspective, a more thorough understanding of the role that betulinic acid has in wound healing should come from the exploration of its effect on macrophage polarization, as the transition of local macrophage populations from a predominantly pro-inflammatory phenotype (M1) to an anti-inflammatory phenotype (M2) is crucial to an effective wound closure Furthermore, Wang et al. noted that betulinic acid mitigated the myocardial ischemia reperfusion injury by oxidative stress inhibition and cell apoptosis due to the inhibition of JNK phosphorylation, p38 by Nrf2 and HO-1activation [37].

Bai et al. conducted an in vivo study on male Sprague–Dawley rats, focused on investigating the effect of betulinic acid on lipopolysaccharide-induced vascular hyporeactivity on an aortic contraction-relaxation model [38]. Betulinic acid downregulated the pro-inflammatory factors TNF-α and IL-1β triggered by the LPS treatment thus exhibiting a potent anti-inflammatory effect.

In a study regarding the viability of skin flaps on mice, betulinic acid increased de number of micro vessels, diminished the tissue edema and sustained the survivability of the skin flap [39]. Western blotting demonstrated an increase in the proteins related to angiogenesis (cadherin 5, VEGF and MMP9) after betulinic acid treatment while the CASP3 level, for the vessels and stromal cells, decreased compared to the control group. In terms of oxidative stress evaluation, an important upregulation for HO-1, eNOS and SOD1 proteins was observed; hence, betulinic acid was able to promote angiogenesis, lower the oxidative stress, and reduce apoptosis, through autophagy [39].

Ebeling et al. conducted a complex study, in vitro on human keratinocytes and ex-vivo on pig ear, in order to establish the wound healing effect of birch bark extract compared to individual isolated triterpenes (betulin, betulinic acid, lupeol, oleanolic acid and erythrodiol) [40]. For the porcine excision wound healing model, an oleogel containing 10% birch bark extract and 90% sunflower oil was used; after 48 h, a faster reepithelization process occurred. The same 10 µg/mL extract was dissolved in PBS and applied in the same wound model resulting in a similar effect evaluated after 48 h and consisting in the acceleration in wound healing as compared to the plain PBS. In addition, the same concentration of betulin found in the extract (8.69 µg/mL) was also applied to the wound, but the effect was lower compared to the extract, thus supporting the conclusion that the other associated extract components significantly contributed to the recorded result. Betulin and birch bark extract promoted the formation of the skin barrier, also a very important phase in the complex process of skin restoration [40]. When applied on human keratinocytes, the effects of betulin as well as the corresponding extract influenced the inflammatory stage by increasing COX-2 (cyclooxygenase-2), IL-6 (Interleukin 6) and IL-8 (Interleukin 8) levels. Also, the birch bark extract, lupeol, betulin and erythrodiol affect the actin cytoskeleton by stimulating the actin filopodia, stress fibers and lamellipodia formation, dependent on Rho GTPases activation [40].

#### 3.1.2. Lupeol

Lupeol is a triterpene found in many edible fruits and vegetables and in various plant species, standing as a major constituent in medicinal herbs from the Fabaceae or Euphorbiaceae families with demonstrated anti-inflammatory, anti-oxidant, anti-diabetic, anticancer, and hepatoprotective effects [41]. Baserra et al. conducted an in vitro migration assay in order to assess the effect of lupeol on wound healing (proliferation, migration, cell contraction) and also to elaborate on a possible mechanism of action [42]. Lupeol was extracted from *Bowdichia virgilioides* using a 95% alcohol solution as solvent, and then isolated using multiple purification steps; when lupeol was used in high concentrations, the cell proliferation decreased in fibroblasts and also in keratinocytes, without affecting cell viability. Also, the wound healing increased in keratinocytes and the contraction of dermal fibroblasts progressed in the collagen gel matrix [42]. Moreover, the level of p38 and Akt proteins, involved in cell apoptosis and tissue repair, was significantly increased while Tie-2, receptor of the vascular endothelial cells involved in angiogenesis, decreased in a dose-dependent manner; also, an increase of p-Tie-2 levels (the phosphorylated isoform) was recorded. Hence, the anti-inflammatory activity of lupeol could be explained by the suppression of NF-κB (nuclear factor kappa-light-chain-enhancer of activated B cells) expression in keratinocytes and the regulation of MMP-2 (matrix metalloproteinase-2) [42]. One year later, Baserra et al., using the same extract (*Bowdichia virgilioides*), conducted an in vivo study on rats aiming to evaluate the healing properties of lupeol in wounds generated after streptozotocin-induced hyperglycemia on an excision wound model [43]. Lupeol was topically applied as cream on the wounds and the histopathological examination indicated an enhanced vascularization and proliferation of fibroblasts and deposition of collagen fibers. The ELISA assay revealed an upregulation of the IL-10 levels (anti-inflammatory cytokines) and a downregulation of the IL-6 levels (pro-inflammatory cytokines). Moreover, an enhanced intensity of collagen III production, TGF-β1 (transforming growth factor beta 1) and FGF-2 (basic fibroblast growth factor) as well as a repression of NF-κB were reported. While this research provides important elements regarding the effects of lupeol on wound healing, additional research employing human models are needed. Significant differences—wound contraction in rats as compared to re-epithelization in humans—underline the importance of further studies in humans.

Furthermore, lupeol counteracted the oxidative stress and increased Sod-2 (superoxide dismutase 2) and Ho-1 (hemeoxygenase 1) levels [43].

Research also revealed that lupeol has an anti-aging effect on UVA aged fibroblasts [44]. It is well-known that repeated UVA skin damage can produce DNA alteration, ROS generation and could also affect MMPs expression. The study revealed that lupeol treatment on UVA radiated dermal fibroblast inhibited p16, p21 and p-p53 and decreased the MMP-1, -2, -3 expression.

In summary, lupane-type triterpenes exibit a notable anti-inflammatory effect via the NF-*κ*B signaling pathway and the supression of pro-inflammatory cytokines. The triterpenes present in birch bark demonstrate an improved re-epithelization in porcine ear—a model which is recognized for human cutaneous wound repair due to anatomic and physiologic similarities, as opposed to rodent models.

### 3.2. Ursane-Type Triterpenes

**Figure 4:**
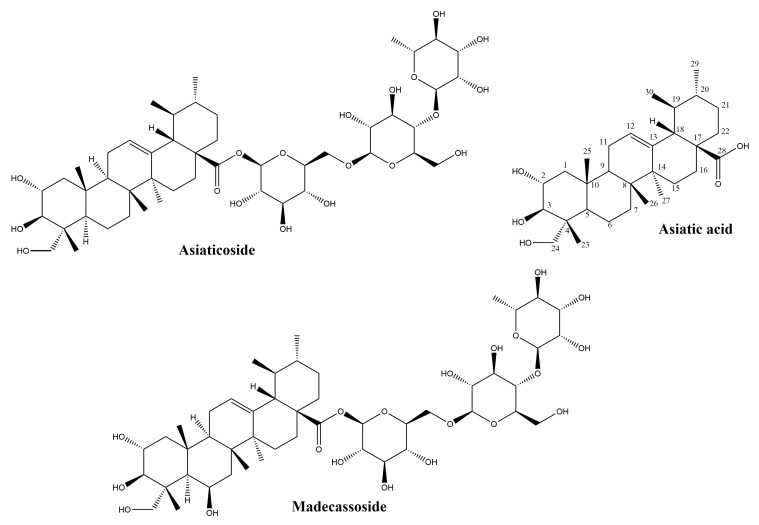
Chemical structures of ursane-skeleton triterpenes.

The main source of ursane-derived triterpenes (Figure 4) with high efficiency in the treatment of wounds is Indian pennywort, *Centella asiatica* (L.) Urb. (syn. *Hydrocotyle asiatica* L.), a plant of Asian origin belonging to the Apiaceae family. Its leaves contain triterpenes of the ursane series (asiaticoside, asiatic acid, madecassoside, madecassic acid) and the oleanane series, as well as other secondary metabolites like chlorogenic acids, flavonoids, sterols and essential oils [45].

#### 3.2.1. Asiaticoside

Asiaticoside, the major constituent of *Centella asiatica* extracts, has a glycosidic structure and is made up of the aglycone asiatic acid and three sugar moieties. Its presence in the plant is related to mutualistic interactions with fungal partners: the compound has been isolated from one of the plant’s endophytes, *Colletotrichum gloeosporioides* [46] and its content is enhanced by the fungus Piriformospora indica [47]. Asiaticoside has been intensely studied in the last decade due to its potential in the prevention and treatment of Alzheimer’s disease [48], its neuroprotective effect [49], as a possible novel drug in bone diseases [50], multiple sclerosis [51], allergies [52], cancer [53] and other conditions. The cicatrizing effects of this triterpene have been studied for over six decades [54] and its intervention in nearly all stages of wound healing have been demonstrated. The capacity of asiaticoside to increase the levels of several cytokines including monocyte chemoattractant protein-1 (MCP-1), vascular endothelial growth factor (VEGF), and interleukin-1β is relevant for the inflammatory phase of wound healing, a phase which prepares the migration and division of cells involved in cicatrization [55]. Recently, the effects of asiaticoside on cytokine (tumor necrosis factor-α, IL-6, IL-1β, VEGF) production has been evaluated in an experimental model of rat skin flaps. The increased levels of VEGF were determined through immunohistochemistry and correlated with the improved microcirculatory flow and viability of the skin flaps [56]. VEGF stimulation is crucial for the formation of new blood vessels or angiogenesis during the proliferative phase, allowing the access of oxygen and nutrients to the fibroblasts. Increased VEGF expression by asiaticoside has also been demonstrated in the treatment of deep burn injury for coaxially electrospinning nanofibers loaded with this triterpene [57]. The efficacy of asiaticoside in wound healing was evaluated recently in association to a NO-generator on a diabetic wound model. The mechanism of action indicated the regulation of the Wnt/β-catenin signaling pathway and raised levels of expression for VEGF, CD34, iNOS and eNOS [58].

During the proliferative phase, fibroblasts migrate into the wound and proliferate, then deposit ground substance and collagen. The effects of asiaticoside on events during this phase have been intensely researched [59]; the main effects are promotion of migration and proliferation of normal fibroblasts and stimulation of collagen biosynthesis [60]. Asiaticoside increases the synthesis of type I and III collagen [61]; the mechanism involves activation of the Smad pathway, with asiaticoside binding to Smad 2, Smad 3 and Smad 4. However, asiaticoside is also able to induce collagen synthesis by triggering the transforming growth factor beta receptor I (TβRI) kinase-independent Smad pathway [62]. An additional element in understanding the mechanism for enhanced collagen I synthesis by asiaticoside is offered by molecular docking experiments that identified a possible binding site of this triterpene at the hydrophobic groove of protein phosphatase-1 catalytic subunit (PP1c) [63]. Protein phosphatase-1 (PP1) is an enzyme with an important part in the preservation of homeostasis in keratinocytes [64]. In an in vivo experimental excision wound model, the application of an extract standardized to 2.4% asiaticoside could demonstrate a highly significant shortening of the epithelization period from 10.75 ± 0.50 days to 8.50 ± 0.58 days, after the application of a gel containing 2.5% extract. This observation was correlated with an increase in hydroxyproline content, induction of collagen synthesis, and several markers of wound healing in the histopathological examination: keratinization, fibroblastic proliferation, and neovascularization [65]. Regarding the effects of *Centella asiatica* triterpenes on cellular migration, a research on HaCaT keratinocytes could point out a pro-migratory effect, the stimulation of filopodia formation, and the upregulation of signaling pathways involved in wound healing: FAK, Akt, and MAPK [66].

Interestingly, asiaticoside has the advantage of not only promoting wound healing, but also normalizing cicatrization. It is one of the few natural compounds reducing keloid formation; in fact, it reduces the proliferation of keloid fibroblasts and the synthesis of type I and type III collagen in vitro [67]. The exact mechanism by which asiaticoside is able to reduce the proliferation of keloid fibroblasts, while it promotes the proliferation of normal fibroblasts is not yet understood, and further research is required to explain this paradoxical effect.”

Moreover, this triterpene glycoside is able to reduce the invasive growth of keloid fibroblasts by inhibiting the growth differentiation factor-9/mitogen-activated protein kinase/Smad pathway [68]. In a comparative study evaluating the effects of asiaticoside, alpha-chymotripsin and collagenase on an excision wound model, the use of an ointment with asiaticoside could achieve accelerated wound healing, prevent keloid formation, and a nearly invisible scar formation [69].

The wound healing activity of asiaticoside is linked as well to its antioxidative effect; it has been shown to increase the expression levels of superoxide dismutase [56], to enhance the antioxidant activity of wound catalase, glutathione peroxidase, superoxide dismutase, and to raise the concentration of ascorbic acid, vitamin E and reduced glutathione [70]. The increase in antioxidants is significant in the initial stages of wound healing; it has been proposed that the augmentation of vitamin C levels in the wound tissue is a contributing factor to the raised collagen synthesis [70]. A beneficial effect of asiaticoside in the treatment of wounds is its anti-nociceptive activity; this effect has been determined after oral administration in mice and is mediated through the vanilloid and glutamatergic systems, but does not involve the opioidergic system [71].

#### 3.2.2. Asiatic Acid

Asiatic acid, the aglycone of asiaticoside, has been less researched than its glycoside in the context of wound healing. In standardized extracts of *Centella asiatica*, its content is generally 30%, while asiaticoside represents 40% and madecassic acid 30% [59]. Asiatic acid has been identified in over 40 other plant species, among them common plants like chicory (*Cichorium intybus*, whole plant), basil (*Ocimum basilicum*, whole plant) and pomegranate (*Punica granatum*, seeds) [72]. In vivo, asiaticoside is converted to asiatic acid; pharmacokinetic investigations showed that after the separate administration of equimolar doses of the glycoside and its aglycone, similar plasmatic concentrations of asiatic acid are obtained, albeit these levels are attained later in case of asiaticoside [73]. The absolute bioavailability of asiatic acid after oral administration may be considered low, of only 16.25%, and is related to its poor solubility and rapid metabolism [74].

Earlier studies performed on human dermal fibroblasts suggested that asiatic acid and asiaticoside enhance the synthesis of collagen I in a similar proportion, and that the presence of the glucidic moiety is not mandatory for this effect [75] More recently a comprehensive study aimed at identifying the active triterpene from *Centella asiatica* in wound healing compared the effects of asiaticoside, asiatic acid, madecassoside, and madecassic acid on fibroblast proliferation, collagen synthesis and degradation, as well as after oral administration to mice with experimental burn injuries [76]. The stimulation of fibroblast proliferation could not be pointed out for any of the four compounds. The findings regarding the effects on collagen synthesis in skin fibroblasts were unexpected: only the glycosides (asiaticoside and madecassoside) enhanced this process, as demonstrated by the elevation of mRNA levels for collagen I and III and higher procollagen type I and type III content. Madecassoside proved to be more active than asiaticoside in this regard. Moreover, madecassoside was the only compound able to enhance the expression of metallopeptidase inhibitor 1mRNA expression, a protein which inhibits the collagen degradation. Regarding the effects on the TGF-β/Smad pathway, highly relevant to collagen synthesis by fibroblasts, only the triterpene glycosides were able to activate this pathway with madecassoside being more active. The advantages seen in vitro of glycosides over aglycones were supported by in vivo experiments, where oral administration of triterpenes to mice with burn injuries only induced healing activities in case of the glycosides; madecassoside was more efficient than asiaticoside. A possible explanation for these observations, postulated by the authors, is that the glycosides have a particular absorption and distribution rate in the state of burn lesions [76].

*Centella asiatica* is acknowledged for reducing keloid formation and is currently being used in scar management [77]. The development of keloid during cicatrization is due to excessive deposition of extracellular matrix during wound healing, with the formation of unaesthetic scars; it is believed that transforming growth factor (TGF)-β/Smad pathway is a key element in keloid genesis. In order to evaluate the effects of asiatic acid on keloid, both normal and keloid fibroblasts were exposed to TGF-β1 in the presence or absence of asiatic acid. In keloid fibroblasts, the investigated compound could inhibit TGF-β1 stimulated expression of collagen and plasminogen activator inhibitor-1; the observed inhibition implied PPAR-γ activation [78]. Asiatic acid also has antinociceptive and anti-inflammatory effects. In an assay evaluating the analgesic effect of asiatic acid in mice on a hind paw edema model using the acetic-acid writhing response and the formalin-induced licking experimental settings, the analgesic effect was similar to that of indomethacin used at the same dose (10 mg/kg). The anti-inflammatory effect was correlated to a reduction of the seric levels of malondialdehyde, inducible nitric oxyde synthase (iNOS), tumor necrosis factor alpha and interleukin 1β. These findings were in agreement with the inhibition of iNOS, cyclooxygenase-2, and nuclear factor NF-κB proteins expression. Moreover, the activities of catalase, superoxide dismutase and glutathione peroxidase in hepatic tissue were significantly reduced, demonstrating a protective activity against reactive oxygen species [79].

#### 3.2.3. Madecassoside

This compound is a structural analogue of asiaticoside, differing by the presence of a hydroxyl group. Unlike asiaticoside, it may be absorbed intestinally; madecassoside and madecassic acid could both be detected in rat plasma after oral administration of the glycoside, with inflammatory status significantly affecting their seric levels [80]. The compound is a major triterpene in *Centella asiatica*, and its wound healing effects have been pointed out both in vitro and in vivo; it is able to intensify the proliferation of normal fibroblast, to boost collagen synthesis by activating the TGF-β/Smad pathway, and to reduce collagen degradation [68]. When administered orally on a burn wound model, madecassoside helps to achieve faster wound closure, reduces infiltration of inflammatory cells and enhances epithelization. These effects are linked to antioxidant properties decreasing malondialdehyde and nitric oxide while increasing glutathione and hydroxyproline concentrations and promoting angiogenesis [81]. Madecassoside and asiaticoside can both increase the secretion of chemoattractant protein-1 production and favor wound contraction in burn wound models [82]. Madecassoside-loaded liposomes with enhanced bioavailability applied locally were shown to be efficient in the healing of burn wounds [83].

In conclusion, ursane-type triterpenes are perhaps the most intensely investigated triterpenes in the context of wound healing, mostly due to the ethnomedical background of *Centella asiatica*, the main source of these compounds. Asiaticoside, the main constituent the plant, has the advantage of not only promoting wound healing, but also normalizing cicatrization as it reduces keloid formation. Anti-inflammatory effects, enhanced collagen synthesis in normal fibroblasts and stimmulation of angiogenesis are the main contributors to the wound healing effect of these compounds.

### 3.3. Oleanane-Type Triterpenes

#### 3.3.1. Oleanolic Acid

Oleanolic acid is largely present in the plant kingdom, with over 120 species serving as natural sources [17]. The cicatrizing effect was highlighted in a research performing a bioactivity guided fractionation of a plant used in traditional Peruvian medicine, *Anredera diffusa* (Moq.) Sperling (Basellaceae). The authors documented the accelerated closure of wound and enhanced tensile strength on an excision wound model [84].

Oleanolic acid also enhanced wound closure through the stimulation of the migratory activity of mice fibroblasts [85]; several researches showed that oleanolic acid displays gastroprotective effects in vivo on induced gastric ulcer models [86,87,88]. The stimulating effect of oleanolic acid on cell migration could also be noted on two epithelial cell lines (Mv1Lu and MDA-MB-231) subjected to scratch assays; the underlying mechanism involves the stimulation of pathways involving mitogen-activated protein (MAP) kinases [89]. To the best of our knowledge, oleanolic acid has not been directly investigated in burn wounds.

#### 3.3.2. Glycyrrhizin

This is an oleanane-type triterpene saponin from the underground parts of licorice, *Glycyrrhiza glabra* L. This compound has some remarkable properties in the context of wound healing. Glycyrrhizin inhibits high-mobility group box 1 protein (HMGB1), secreted by immune cells including macrophages; this property explains its anti-inflammatory effects on thermal injuries models [90] as well as its antifibrotic effects on keloid fibroblasts, acting as a cell death regulator [91]. In fact, the inhibition of HMGB1 by glycyrrhizin has been shown to inhibit pro-inflammatory M1 phenotype, in parallel with anti-inflammatory M2 phenotype activation in microglia/macrophages [92]. The investigation of glycyrrhizin’s effect on the M1/M2 transition of skin macrophages may shed additional light on its mechanism in wound healing.

Several other oleanane-derived triterpenes with activities relevant for wound healing have been isolated from plants: camellioside B is a major constituent of *Camellia japonica* L. flower buds, able to enhance fibroblast proliferation and to inhibit melanogenesis in vitro [93]. Ten oleanane derivatives identified in *Bellisperennis* L., a plant with cicatrizing effects from the Asteraceae family, have been shown to promote collagen synthesis in vitro; these derivatives include six types of perennisosides (I, II, VII, IX, XI and XVIII), asterbatanoside D, bernardioside B2, and two bellissaponins (BS5 and BS9) [94]. One of the most widely-used cicatrizing medicinal plant is marigold, *Calendula officinalis* L. [95]; its flowers contain between 2–10% derivatives of oleanolic acid, divided in two families: 3-O-monoglucoside and 3-O-monoglucuronide derivatives [95,96]. Marigold extracts act during the inflammatory phase by activating nuclear factor NF-κB transcription and triggering high levels of interleukin 8 in keratinocytes; while no pro-migratory effects were recorded, the inhibition of collagenase increased the collagen quantity) [97].

**Figure 5:**
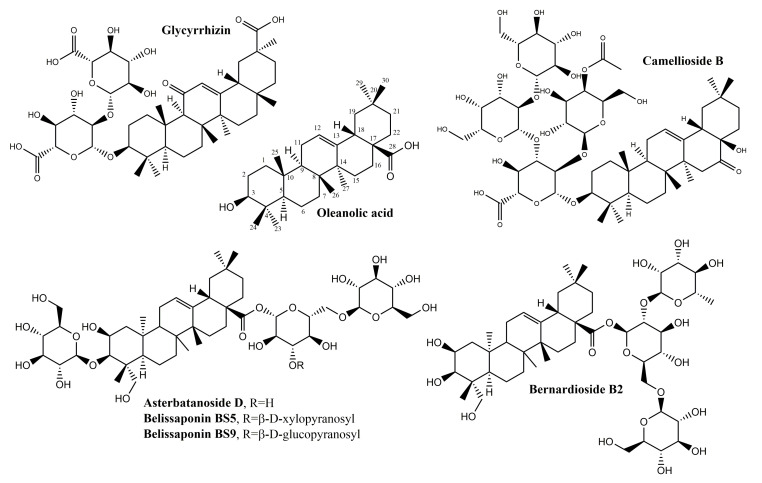
Chemical structures of oleanane-skeleton triterpenes A.

**Figure 6:**
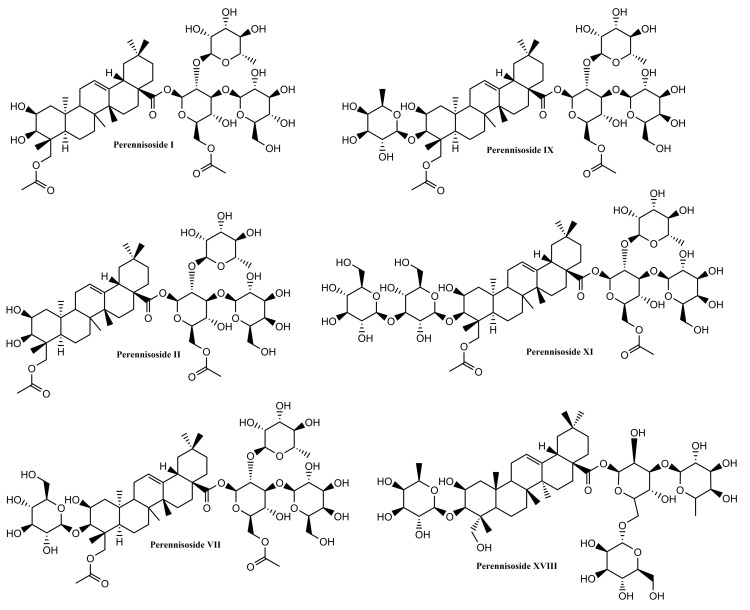
Chemical structures of oleanane-skeleton triterpenes B.

In the class of oleanane-type-triterpenes (Figure 5 and Figure 6), available evidence underlines the potential of glycyrrhizin in the area of wound healing. Its modulating effect of macrophages via the inhibition of high-mobility group box 1 protein (HMGB1) makes it an interesting research target in chronic wounds. Oleanane triterpenes from marigold, a reputed plant with wound healing effects, deserve additional interest for a better undersanding and use of its constituents.

**Figure 7:**
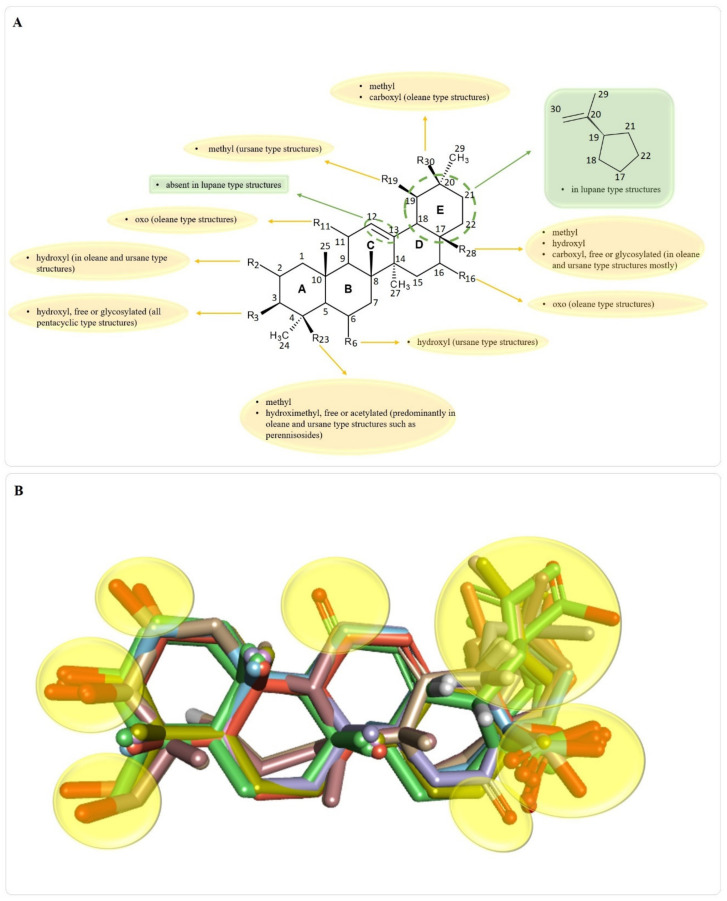
Key structural features present in all pentacyclic triterpene structures with wound healing properties (**A**); superimposed 3D structures of wound healing pentacyclic triterpene structures (**B**); areas where key functional groups appear are highlighted in yellow; structures that have glycosylated OH or COOH groups, are depicted only as triterpene scaffolds for better image clarity.

As stated, there is a wide variety of pentacyclic triterpenes with wound healing properties. The structural differences regarding the basic scaffold of the above-mentioned molecules represent key elements that can lead to a better, structure-activity relationships, in developing/testing new compounds with wound healing potential. Different structural features present in pentacyclic triterpene structures with wound healing properties, are depicted in Figure 7.

### 3.4. Dammarane-Type Triterpenoids

**Figure 8:**
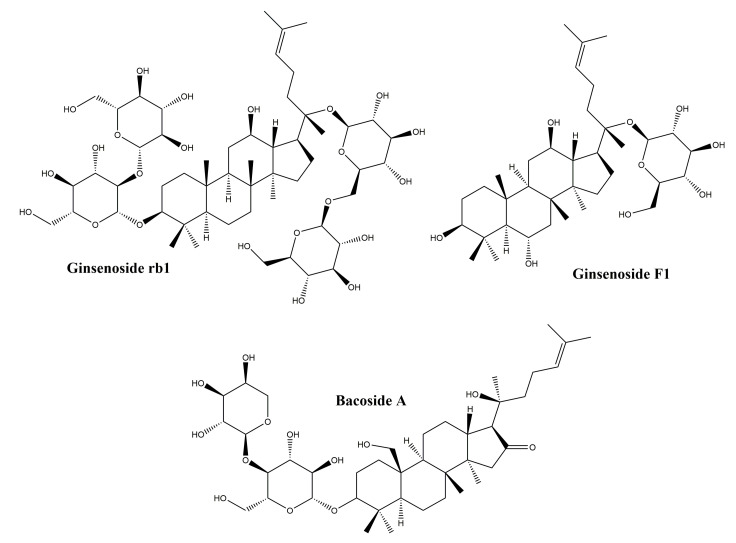
Chemical structures of dammarane-type triterpenes.

Dammarane-type triterpenes belong to the class of tetracyclic triterpenes; they represent a wide range of compositions spread in a variety of plant families such as Arialiaceae, Betulacease, Meliaceae, etc. [98]. Their basic structure is represented in Figure 8.

#### 3.4.1. Ginsenosides

The major representatives of the dammarane-type triterpene class are the ginsenosides. In terms of chemical structure, ginsenosides can be grouped into two classes: protopanaxadiol and protopanaxatriol glycosides. The main plant source of ginsenosides is ginseng, belonging to the *Panax* genus. Ginsenosides are widely used in traditional Chinese medicine for their tonic effect, but also in traditional medicine for their numerous pharmacological effects such as immunostimulatory, antitumor and antihypertensive [99]. In addition, ginsenosides are also known for their biological effects such as: anti-inflammatory activity by reducing the production of nitric oxide, Cox2 and proinflammatory cytokines [100], regulation of cell proliferation, differentiation and apoptosis [101] and regulating migration, invasion, and angiogenesis in various cell types [102]. Following the observation that ginsenosides increase cell proliferation in different healthy cell types, while inhibiting proliferation in tumor cell types [103], numerous studies have focused on their healing effect, trying to explain their mechanism of action. In addition, in vitro and in vivo studies performed on purified ginsenosides, but also on ginseng root extracts, have shown that they have a beneficial effect on skin lesions [104,105,106]. Although there are numerous studies that highlight the repairing role of ginsenosides, the mechanism of action is not fully elucidated. In a study by Lee et al., the protective effect of some ginsenoside derivatives on HaCaT human keratinocyte cells exposed to UVB ratios was observed [104]; it has been shown that ginsenosides reduce cell death after exposure to UVB radiation. After treatment with ginsenosides and exposure to UVB radiation, it was reported that they prevent a decrease in Bcl-2 mRNA expression, concluding that ginsenosides have a protective effect on keratinocytes by maintaining a constant level of Bcl-2 [104]. In another in vitro study by Lee et al., on human dermal fibroblasts, results showed that ginsenosides obtained from a *Panax ginseng* root extract exert a healing effect by increasing type I collagen synthesis through the activation of the Smad pathway; the antioxidant effect of the extract also contributes to the healing process [107].

In a more in-depth study of the mechanism of action of ginsenosides by Shin et al. [108] it has been reported that the healing effect of ginsenosides is related to their property of increasing the migration of human keratinocytes through a sphingosine-1-phosphate (S1P) dependent mechanism. The data obtained by Shin et al. support the hypothesis that ginsenosides act through the S1P pathway, noting that they increase S1P production by regulating the activity of enzymes associated with the generation and degradation of S1P [sphingosine kinase 1 and S1P lyase). Another possible mechanism of action associated with ginsenosides was explained by Kimura et al. in an in vivo study on burn wound healing in mice. The repairing effect of Red Ginseng root extract has been associated with increased neovascularization around the wound and the production of vascular endothelial growth factor and inteleukin-1β [106]. Hence, the repair effect of ginsenosides has been tested on various in vitro and in vivo models, but the mechanism of action is not yet fully elucidated; in addition, the possible side effects associated with their use are not well-known. For these reasons, more studies are needed, also performed on human subjects, in order to fully demonstrate the effect of ginsenosides in wound healing.

#### 3.4.2. Bacosides

The bacosides are part of the class of saponin triterpenes which are found predominantly in the extract of *Bacopa monnieri*, from the Scrophulariaceae family [109]. They are known for their therapeutic effects demonstrated in numerous studies, such as: the antioxidant effect and the potential to inhibit the production of superoxides [109], the anti-metastatic potential in hepatocellular carcinoma [110], as well as the cicatrizing effect on skin lesions and the potential to accelerate wound closure [111]. In an in vivo study by Sharath et al., the repair effect of bacosides vs. total methanolic extract of *Bacopa monnieri* was evaluated on incision wound models; results showed that bacosides and methanolic extract exhibit a repairing effect similar to the synthetic drug nitrofurosone used as control. The effect on wound healing is presumably due to the astringent and antibacterial effect exerted by bacosides; it has also been noticed that by oral administration of bacosides, collagen deposition in the affected tissue is increased, thus increasing tissue resistance and healing [111]. Another in vivo study was performed in order to evaluate the effect of bacosides on the wound healing caused by burns in rabbits; after 28 days of topical application of a bacoside A extract, the levels of collagen, hydroxyproline and hexosamine decreased, as did the size of the wound. Authors hypothesized that the healing effect of bacoside A is due to a complex mechanism of action that involves: (i) changing the structure of collagen fibers; (ii) upregulation or downregulation of beta-1 growth factor and metalloproteinase-1 matrix transformation [112].

Collectively, dammarane-type triterpenoids such as ginsenosides and bacosides were shown to improve wound healing by type I collagen synthesis, increasing neovascularization and cytoprotective effects. Hence, dammarane-type triterpenes might contribute to human skin healing process especially in the proliferation stage, by increasing collagen synthesis and neoagiogenesis or by preventing infection developpement of the wounded area and speeding up the healing process by exerting astringent and antibacterial effects. Nevertheless, further experiments on humans are necessary to explore their potential in re-epithelization

### 3.5. Lanostane-Type Triterpenes

The group of lanostane-type triterpenes includes several compounds that have a common skeleton represented by a structure of tetracyclic origin [113]. They are found in many plant sources, mainly in mushrooms; the largest number of lanostane-type triterpenes have been isolated from species of the Polyporaceae family [114].

Lanostane-type triterpenes have many demonstrated pharmacological properties such as antitumor [115], hepatoprotective [116] and anti-inflammatory [117] activities. Some representatives of the lanostane group also have beneficial effects on carbohydrate [118] and lipid metabolism [119] and also exhibit anti-infective properties [120,121].

#### Cycloastragenol

**Figure 9:**
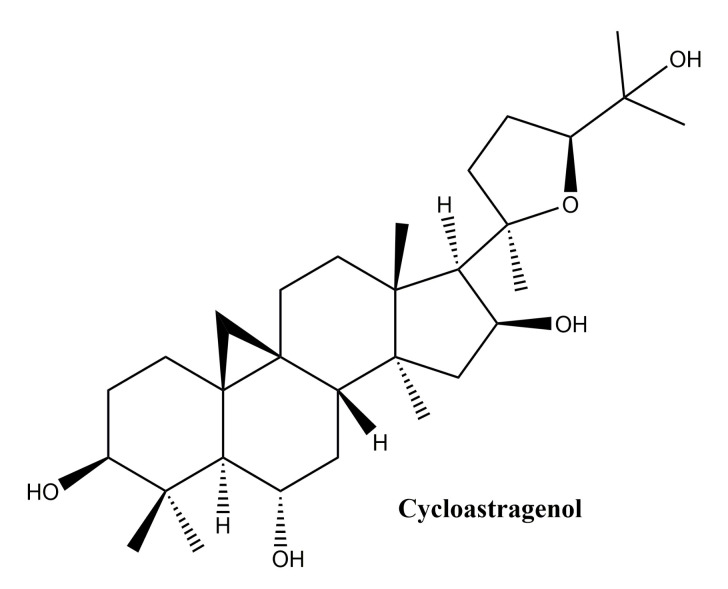
Chemical structure of cycloastragenol, a lanostane-type triterpenes.

This compound (Figure 9) one of the main members oflanostane-type triterpenes and the main active ingredient in *Astragalus membranaceus*, was used for thousands of years in Chinese medicine for its hepatoprotective, immunostimulatory, diuretic, antihypertensive, and antiaging properties [122]. In a study by Shih-Yu et al. on the effects of cycloastragenol on wound healing, its effects on human keratinocyte cell lines, human dermal fibroblasts and murine wound healing models were studied. In vitro, cycloastragenol stimulates the activity of the epidermal growth factor receptor; in addition, cycloastragenol stimulates extracellular signal-regulated kinase activity, thus stimulating cell proliferation and migration. In vivo, the beneficial effect on wound healing was observed on both sterile and infected wound model; the mechanism of action underlying the in vivo therapeutic effects has been associated with the stimulation of angiogenesis in damaged tissues [123]. Cycloastragenol, the hydrolysis product of *Astragali Radix*, with saponin structure, was reported to stimulate telomerase activity as well as cell proliferation in human neonatal keratinocytes [124]. Based on these observations, Cao et al. conducted an extensive in vitro study to observe the mechanism of action of cycloastragenol associated with wound healing; the main results can be summarized as follows: (i) cycloastragenol stimulates human epidermal stem cell proliferation and migration—EpSCs; (ii) there is an increase in reverse transcriptase telomerase expression which is involved in different types of lesions, by activating specific signals, such as NF-kappa B and autophagy; (iii) the Wnt/β-catenin signal activation, with well-known implications for organ development, stem cell growth, maintaining epithelial turnover during homeostasis and in response to injury [125].

In conclusion, lanostane type triterpenes may influence wound healing in human skin by intervening in proliferation by angiogenesis promotion, also, they could enhance the reepithelization process by influencing proliferation and migration of epidermal cells.

### 3.6. Cycloartane-Type Triterpene

**Figure 10:**
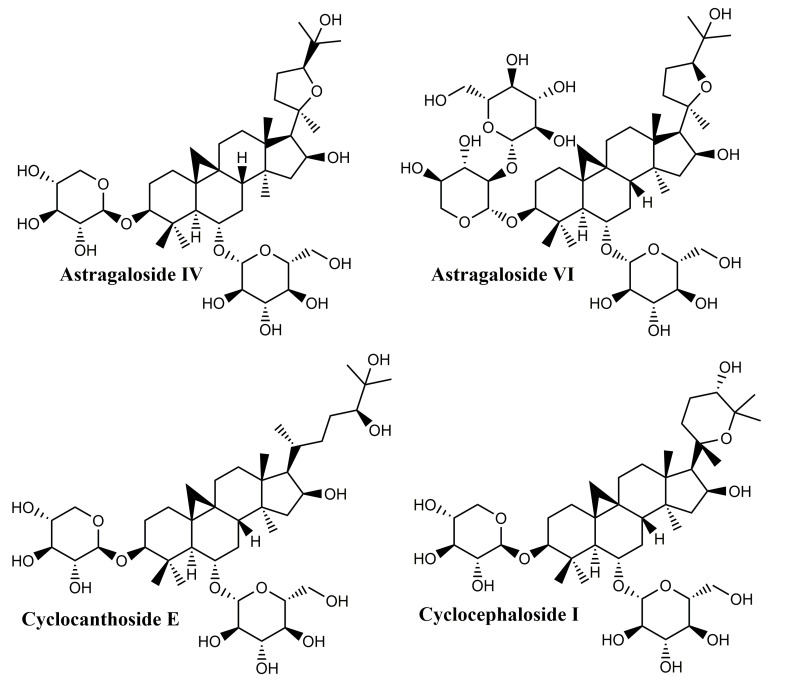
Chemical structure of cycloartane-type triterpenes.

Cycloartane triterpenoids (Figure 10) are widely distributed in various plants but can be found in particular in Astragalus (Fabaceae), Cimicifugea and Thalictrum (Ranunculaceae) genera; the first two mentioned genera contain some of the most popular species used in traditional Chinese medicine [126]. Cycloartane triterpenoids, with unique complex structures, have aroused increased interest in the last decade due to their diverse bioactivities; a quick search in the PubMed database has revealed 251 articles published in the last ten years which indicate their potential as new lead compounds in the medical field. A comprehensive review of the structures and activities of cycloartane triterpenoids and glycosides was published in 2013 indicating their hypolipidemic, hypotensive, diuretic, anti-inflammatory, sedative, analgesic, immune stimulating, and cardiotonic activity [127]. In particular, the antibacterial properties, manifested against Staphylococcus aureus and vancomycin-resistant Enterococcus faecium, as well as the anti-inflammatory and immune-enhancement effects of cycloartane-based triterpenes are significantly contributory factors in their wound healing activity [126].

Their structure is based on the 9b,19-cyclo-5a-lanostane (cycloartane) (I) skeleton and the first structurally characterized representative was cycloartenol, obtained by the reduction of the corresponding ketone, cycloartenone. The majority of these compounds occur in plant sources as esters of organic acids as well as glycosides; biogenetically, they are precursors of phytosterols, occurring only in photosynthetic eukaryotes and contributing to the synthesis of cell membranes [127]. Four main saponins isolated from *Astragalus* species were found to have wound healing effects: cycloastragenol, astragaloside IV, cyclocephaloside I and cyclocanthoside E; their activity was assessed through MTT assay on human keratinocytes as well as proliferation and migration scratch assay [128]. The in vitro studies were supplemented with in vivo incision wound studies on Sprague–Dawley male rats; all compounds showed increased fibroblast proliferation and migration, with clearly superior effects for cycloastragenol at 1 ng/mL. Overall, the study proved that cycloartane-type saponins are the main active compounds isolated from the roots of *Astragalus* species directly responsible for their well-known wound healing activity.

Due to its wound healing promoting activity, astragaloside IV, the major bioactive compound found in *Centella asiatica* (Umbelliferae), was formulated as hydrogel based on sodium alginate-gelatin thus achieving a sustained release at the wound site [129]. When tested on the rat skin excision model, the hydrogel inflicted the significant improvement of collagen synthesis and skin tensile strength recovery through the activation of skin appendages regeneration and the increase of the transforming growth factor-β1 (TGF-β1) serum level; overall, a faster wound closure was reported compared to control. Similar results were reported by Chen et al. in 2012 on a skin excision wound model; in addition, anti-scar effects were recorded, presumably due to decreased levels of collagen I/III and a dose-dependent TGF-β1 secretion by fibroblasts [130]. The same research group prepared solid lipid nanoparticles loaded with astragaloside IV and embedded them into a carbomer hydrogel; the enriched hydrogel was tested in vitro and in vivo in terms of wound healing and anti-scar formation effects [131], the study focusing in particular on the re-epithelization, angiogenesis, and extracellular matrix remodeling processes. The nanoformulation exhibited a sustained release of the active compound, increased the wound closure rate and stimulated the angiogenesis and collagen regular organization, thus achieving promising results as wound healing and anti-scar agent.

A more recent study set to evaluate all eight astragalosides isolated from *Astragali radix* both in vitro, on human HaCaT keratinocytes and human dermal fibroblasts, and in vivo on murine models of wound healing [123]; all astragalosides increased the activation of the epidermal growth factor receptor (EGFR) in HaCaT cells, but astragaloside VI showed the strongest effect in this regard. In addition, both astragaloside VI and cycloastragenol-6-O-beta-D-glucoside, which is considered the main astragalosides’ intestinal metabolite, were found to stimulate the activity of the extracellular signal-regulated kinase (ERK) depending on concentration; overall, the EGFR-dependent cell proliferation and migration was reported in both skin cell lines. The in vivo assessment revealed an accelerated healing of the sterile and infected wounds, presumably due to enhanced angiogenesis.

Conclusively, cycloartane-type triterpenes are the active molecules of some Fabaceae and Ranunculaceae plants. Among them, cycloartanes from the roots of Astragalus membranaceus were tested in various in vitro and rodent in vivo models. They displayed effects like the stimulation of skin cell proliferation and migration, increased angiogenesis improvement of collagen synthesis and skin tensile strength. Although preliminary results appear to be encouraging, other experimental settings (porcine cutaneous wound healing model, studies involving human volunteers) should be approached in order to evaluate their wound-healing potential. Even so, cycloartane-type triterpenes could improve wound healing in human patients by intervening in several stages of healing. These phytocompounds might influence proliferation by enhancing fibroblast proliferation, collagen synthesis and angiogenesis. Moreover they could prevent scarring by inducing a regular organization of collagen. Also, they could influence the remodeling phase by accelerating skin tensile strength recovery. In adition, these phytocompunds might promote an overall faster wound closure partialy due to their antibacterial properties. The most representative substances with triterpenoid structure with in vitro and in vivo wound healing activities as well as their specific mechanism of action are included in Table 1 and Table 2, respectively.

### 3.7. Plant Extracts with Complex Composition

Currently, there are numerous studies on isolated phytochemicals and their biological activities including wound healing; however, the first step in employing vegetal compounds for therapeutic purposes was the use of total herbal extracts with complex composition. Several such herbal extracts were found to promote faster wound healing then control groups during in vivo studies; their complex composition included triterpenes in combination with other active agents like alkaloids, flavonoids, tannins, saponins and antraquinones [9]. Despite the fact that pure compounds isolated from plants may have high activity against various pathologies, they may also be less effective compared to crude extracts containing the same concentration in active agents; there are three reasons identified for this phenomenon: (1) in the unrefined extracts their complex composition leads to multiple interactions between components, including synergistic or cumulative biological effects, including enhanced bioavailability, that overall enhance the therapeutic outcome or reduce side effects; (2) a complex composition allows a multi-targeted approach, particularly useful in diseases with multifactorial etiologies; and (3) the raw extracts may contain compounds which inhibit the multi-drug resistance phenomenon. In addition, the low cost of vegetal extracts compared to pure drugs makes them more affordable to the large population, including the poorest countries of the world [132,133,134].

Birch bark has been long known to exert wound healing effects; its composition consists in a mixture of pentacyclic triterpenes, mainly betulin and betulinic acid. Total triterpene extract was tested in vitro and in vivo on an excision wound healing model and revealed the transient up-regulation of pro-inflammatory mediators such as cytokines, chemokines and cyclooxygenase-2, and an enhanced migration of keratinocytes thus addressing both the inflammatory and the second phase of wound healing [40]. *Calendula officinalis* extracts exhibit a complex composition which includes polyphenols, flavonoids, carotenoids and triterpene alcohols, both as free and esterified forms [135]. The flower extract was tested orally and topically on excision wounds in rats and assessed in terms of re-epithelization and wound closure percentage [136]; the percentage of wound closure almost doubled in the presence of the vegetal extract compared to control and the time interval needed for re-epithelization was reduced significantly. Buzzi et al. assessed in 2016 the therapeutic effects of *Plenus dermax*, a bioactive extract of *Calendula officinalis*, in venous leg ulcer healing; the extract contains a mixture of flavonoids, terpene alcohol and triterpenoid monoesters, which are known to act against skin oedema and inflammation and to promote wound healing [137]. The study monitored the wound area in terms of planimetry, infection and clinical status, and revealed a double percentage of complete re-epithelization in treated patients compared to control as well as a 4-fold increase in healing velocity percentage per week.

*Centella asiatica* extracts are mainly composed of several triterpenes whose beneficial effect in the treatment of wounds of various etiology is well known, the plant being used in medicinal and nutraceutical products [138]. A series of vegetal extracts were prepared by using sequential hexane, ethyl acetate, methanol, and water as extraction solvents; the extracts were applied topically, once daily, on incision and partial-thickness burn wound models in rats [139]. All extracts significantly improved the tensile strength of incision wounds and the epithelialization and keratinization of burn injuries; the ethyl acetate extract, containing mainly asiatic acid, exhibited the strongest healing activity. *Antrodia cinnamomea* is a mushroom used for medicinal purposes due to its antitumor, immunomodulating and anti-inflammatory activities; phytochemical studies revealed the presence of polysaccharides, proteins, fatty acids, and phenyl derivatives but mostly triterpenoids [140]. Various concentrations of triterpene ethanolic extract was orally administered to streptozotocin-induced hyperglycemic mice; the assessed parameters such as weight gain, volume of drinking water, liver and spleen weight revealed a positive regulation of metabolic abnormalities. By reducing hyperglycemia, the triterpenes extract was able to modulate the inflammatory response at the wound site and promote wound recovery.

Licorice, *Glycyrrhiza glabra* L., which contains triterpene saponins, flavonoids, isoflavonoids, chalcones and glycyrrhizic acid, displays a large plethora of biological effects; the hydroalcoholic extract and its cream formulation were tested on full thickness wounds in guinea pigs revealing an increase in epidermal formation, collagen deposition and angiogenesis as well as a decrease in acute inflammation thus improving the wound healing rate in a dose-dependent manner [141]. The extract of the roots of *Rubia cordifolia* L. contains mainly antraquinones combined with flavonoids, polyphenols, saponins and triterpenoids (including pentacyclic triterpenes); it has long been used in the Ayurvedic medicine [142]. The ethanolic extract and its formulation as hydrogel were tested on excision wounds in mice; the histological evaluation highlighted a significant infiltration of the inflammatory cells, enhanced angiogenesis and cell proliferation, resulting in wound healing effects and a shortened inflammatory phase [143]. *Emblica officinalis* (Euphorbiaceae) is an important plant in Ayurvedic medicine, containing mainly ascorbic acid, polypenols, flavonoids and tannins [144]; two triterpenes were found in the plant roots: secofriedelanophyllemblicine (seco-friedelane type) and ursophyllemblicoside (ursane-derived saponin) [145]. The alcoholic extract of emblica fruits was tested in vivo on excision wounds and was found to increase cell proliferation and stimulate collagen synthesis at the wound site, presumably through an antioxidant mechanism [146]. The extract of *Jatropha curcas* (Euphorbiaceae) stem bark was assessed in terms of wound healing effect on excision and incision wounds in rats by using standard silver sulfadiazine (0.01%) cream as reference. All monitored parameters—percentage of wound contraction, hydroxyproline content and tensile strength including histopathological studies—showed the significantly superior wound healing effect of the extract ointment compared to control [147].

## 4. Innovative Formulations Containing Wound Healing Triterpenes

The wound healing properties of natural products are exerted through celullar and molecular mechanisms that induce antioxidative stress, anti-inflammatory effects, neovascularisation and angiogenesis, ultimately resulting in re-epithelialization and wound regeneration [148]. Traditionally, natural compounds are delivered to the wound site as topical applications of ointments with various lipophilic/hydrophilic compositions. However in the last decade, numerous innovative topical formulations occurred, enabling their improved efficacy and bioavailability; in addition, through specific technological interventions, such as nanoformulation, phytochemicals can be included in controlled delivery systems with enhanced permeability to more profound skin layers, thus speeding up the healing process [148]. The European Medicines Agency has already approved in 2016 the use of an oleogel containing a dry extract from the outer bark of birch in order to treat partial-thickness skin wounds [149,150]; oleogels with birch bark extract are stable and can incorporate water up to 60%, thus resulting in stable w/o emulsions [151,152]. Compared to oleogels, oil-in-water (o/w) emulsions with birch bark triterpenes do not induce a significant effect towards wound healing thus indicating that the oil used as vehicle has a dual effect: modulates the release of active compounds and directly influences the skin healing process [153].

Färber et al. comparatively assessed oleogels, water-in-oil emulsions and water-in-oil foams containing birch bark triterpenes in terms of their permeation flux, by quantifying betulin permeation through porcine skin; experimental data showed that similar results were obtained from all samples, thus indicating that foams can be successfully used in wound healing [154]. The advantage of foams lies in the fact that, unlike oleogel application which can cause physical stress and pain, foams allow almost touchless application thus leading to a better patient compliance. Nanoemulsions have become frequently used as carriers for the skin delivery of active drugs due to their particle sizes which increase the contact surface area thus exhibiting higher efficacy, safety, permeability and bioavailability compared to other topical formulations [155]. Mixtures of natural or synthetic pentacyclic triterpenes, respectively, were formulated as nanoemulsion by using various ratios of surfactant/co-surfactant mixture and assessed in terms of anti-inflammatory activity by means of Draize test on rabbits; the study revealed a high amount of active compounds retained to the skin level and thus available for local effect as well as strong anti-inflammatory activity and the lack of toxic and/or irritative reactions [156]. In addition, the mixture of natural triterpenes was significantly more effective as anti-inflammatory agent than its synthetic counterpart.

Various hydrogel formulations with *Chlorella vulgaris* extracts were prepared and tested in vivo on Swiss albino mice with induced skin injuries; an antibacterial gel was used as reference [157]. The extracts were characterized in terms of phytochemical profile, exhibiting triterpenes among the active compounds; their topical application revealed pro-healing and anti-inflammatory properties with no atopic or allergic side effects even at high concentrations. Chitosan-gelatin hydrogel films were designed for the entrapment of lupeol, aiming to obtain an ideal wound dressing; in vitro assays revealed a sustained release of lupeol, antioxidant properties as well as lack of toxicity thus qualifying the hydrogel films as promising wound healing formulations [158]. A hydrogel was prepared by tosylating β-cyclodextrin and grafting the product on polyethyleneimine, followed by cross linkage with silk fibroin; the hydrogel was loaded with *Centella asiatica* extract and hydrocortisone and reduced significantly the wound healing time of pressure sores [159]. *C. asiatica* extract, containing a triterpene mixture, was complexed with hydroxypropyl-β-cyclodextrin and formulated as topical spray which showed high efficacy in the treatment of excision wounds inflicted on rats [160]. An asiaticoside-rich fraction was separated from an extract obtained using the *C. asiatica* aerial part and then formulated as hydrogel based on polyvinylalcohol and polyethylene glycol through the freeze-thaw method; the hydrogel was assessed in vivo on rabbits with induced skin injuries by means of the incision model [161]. Experimental results showed that the tested hydrogel accelerated the healing process even compared to commercial creams used as reference while no irritation phenomena were reported.

A step forward in the development of hydrogels was the preparation of nanohydrogels which combine the advantages of hydrogels and nanosystems and avoid the flaws of macroscopic hydrogels such as the fast drug elution from the swollen matrix; nanohydrogels are prepared through intramolecular cross-linking reactions [162]. A nanohydrogel platform was built by using gelatin and glycosaminoglycans to incorporate asiatic acid and metallic nanoparticles, followed by its in vivo assessment in second degree burn wounds in Wistar rats [163]. Results showed a significant wound healing activity through re-epithelization, collagen fibers arrangement and angiogenesis with no apparent toxicity. Nanofibers are fibers displaying diameters within the nanosize range which were developed for multiple purposes including wound healing; in particular, nanofibers obtained by electrospinning show unique properties, such as an ultrathin diameter and high volume ratio while its 3D structure strongly resembles the extracellular matrix [164]. Their very large porosity and gas permeability recommend them as wound dressings; in addition, by functionalization, nanofibers can modulate cells attachment, proliferation and differentiation thus facilitating the skin healing process [164]. Nanofibers can incorporate drugs in order to achieve controlled drug release, increased efficacy and reduced toxicity and side-effects [165]. Ginsenoside, a triterpenoid isolated from *Panax ginseng* extracts [17], was loaded on electro spun poly(d,l-lactide-co-glycolide) (PLGA) nanofibers coated with chitosan through pressure-driven permeation technology; their in vivo assessment was conducted on animal models of experimental wounds and revealed the complete re-epithelization much earlier compared to controls. In addition, scar elevation index measurements and hystological analysis indicated that the application of ginsenoside-loaded nanofibers inhibited scar formation thus showing they also act in the late phases of wound healing [165]. Gelatin nanofibers prepared by electrospinning were loaded with *C. asiatica* extract and then applied on wound models induced in Sprague-Dawley rats; the nanofibers led to the highest skin recovery rate while the presence of the vegetal extracts containing triterpenoids did not influence the hydrophilicity and biodegradability of the fibrous carrier [166].

Metallic nanoparticles, in particular gold, silver and zinc, are nowadays increasingly used in wound healing due to their uniques properties such as the intrinsic antibacterial properties, low skin penetration, easy application, less frequent dressing changes as well as a constantly moist wound environment; in addition, their efficacy and toxicity can be modulated by properly designing their size, architecture, surface functionalization, zeta potential and polydispersity index, by adequately selecting the fabrication method [167]. Silver nanoparticles and the triterpenoid asiaticoside were included in a polyurethane foam synthesized by the reaction between polyols and diisocyanate; hydroxypropyl methylcellulose, chitosan and sodium alginate, respectively, were added to the foam sheets composition [168]. All formulations exhibited antibacterial properties but the foam sheets with alginate revealed the highest release rate of both silver nanoparticles and asiaticoside; in vivo studies on pigs indicated that the alginate-foam dressing improved wound healing both in terms of wound closure as well as histological parameters of the dermal wound without causing any dermatologic reactions [168]. Plant extracts containing triterpenes can also be used as reducing and capping agents for the synthesis of metallic nanoparticles with antibacterial properties; the advantages of the bioreduction methods consist in the use of ecofriendly and biocompatible materials which diminish the risks for biomedical applications [169]. Aazam et al. used in 2016 the aqueous extract of *Ocimum sanctum* (Tulsi) leaves containing ursolic acid among other constituents for the purpose of silver nanoparticles synthesis; the metallic nanoparticles coated with phytochemicals acted as effective Gram positive and Gram negative antibacterial and antifungal agents thus showing potential to reduce infections in burn wounds treatment [170]. A similar approach was reported by Oh et al.who used the aqueous extract of *Chaenomeles sinensis* fruit containing pentacyclic triterpene acids as reducing and stabilizing agents in the synthesis of silver and gold nanoparticles, respectively [169]; the silver nanoparticles thus obtained acted as effective antibacterial agents against Gram positive and Gram negative bacteria.

Nanoliposomes are vesicular nanosystems exhibiting high flexibility, stability and biocompatibility and can therefore be used as dermal delivery carriers with increased drug concentrations delivered into the skin levels as well as longer retention times and efficacy compared to the free drug, traditional liposomes or even ethosomes [171]. Madecassoside, a triterpene found in *Centella asiatica* [17], was formulated as nanoliposomes by a double-emulsion method in order to enhance its transdermal and wound healing effects; the biological effects were clearly superior to those reported for film dispersion liposomes designed by the same research group [83] in terms of leakage rate, stability, absorption and penetration into the skin and encapsulation efficiency. Therefore, madecassoside nanoliposomes stand as promising therapeutic agents for the treatment of a wide range of skin injuries including surgical, ulcers and burns [83].

## 5. Clinical Trials

Despite the well-known potential of medicinal plants in wound healing, a limited number of clinical trials are currently available, assessing the efficacy of various phytochemicals or complex extracts. Although there is a large number of pharmaceutical formulations containing vegetal extracts for the treatment of all type of wounds, some often being used since ancient times, reliable evidences are needed to demonstrate their pharmacological potential in human patients. Hence, an increased number of clinical trials and also the design of more extended studies are crucial for the evidence-based implication of plant phytochemicals, including triterpenes, in wound healing [172].

Ursane-type triterpenes, asiaticoside, asiatic acid, madecassoside, madecassic acid, phytocompounds identified in *Centella asiatica* extracts (CAE), were included in several clinical studies that showed their involvement in improving microcirculation and vascular permeability after oral treatment with either 30 or 60 mg extract in patients with post phlebitic syndrome; they are also able to reduce capillary filtration and edema in patients suffering from venous hypertensive microangiopathy [173]. Also, CAE were demonstrated to reduce stretch marks development in a randomized clinical trial on 80 pregnant women, designed to assess the efficacy of a daily applied topical cream (Trofolastin – novartis, Barcelona, Spain) comparing to placebo; one further observation was that the greatest benefit occurred in women who developed stretch marks in puberty while no benefit was reported in women who acquired them in a previous pregnancy [174]. Moreover, the daily topical application of another cream containing CAE (Alpha Centella, Alphaplast International, Gallo Manor, South Africa) seems to be efficient in reducing scarring during wound healing; the results of a clinical trial on 106 patients for several months highlighted a mechanism of reducing inflammation and increasing type I collagen [175]. A combined treatment with two currently used pharmaceutical formulations containing CAE, Madecassol ointment and capsules, was documented in a clinical trial that enrolled 12 patients suffering from systemic sclerosis and localized scleroderma for 6 months; the study revealed the effectiveness of these products in improving digital ulcerations, and the appearance and coloration of the skin [59]. The same products, as ointment and powder formulation, combined with asiaticoside in i.m administration showed rapid healing outcomes in 12 patients receiving skin grafts and monitored for one week. Meanwhile, their capacity to promote wound healing in ulcerative lesions was demonstrated in a clinical trial that enrolled 98 patients that also underwent a combined topical and i.m treatment, respectively; a cleansing effect on superficially necrotic ulcerations and good perfusion on the wounded area were reported [176]. A randomized clinical trial that included 200 diabetic patients assessed asiaticoside in terms of effectiveness in diabetic wound healing; after daily treatment with 300 mg asiaticoside, the authors concluded that wound contraction was improved and hypertrophic scars and keloids formation was efficiently prevented compared to placebo [177]. Dermatocosmetological studies conducted on CAE demonstrated an antiaging effect mainly due to their content in madecosside, through enhancing type I collagen; in a double blind clinical trial that monitored 20 women with photoaged skin for 6 months the general outcome was an important improvement in the firmness, elasticity and hydration of the skin [178]. Liposclerosis, or cellulite, is another condition that benefits from CAE treatments; a randomized clinical trial that enrolled 35 women orally treated with 60 mg CAE for a period of 90 days, showed reduction in the diameter of adipocytes and inner adipocyte fibrosis [178].Furthermore, madecassoside in combination with panthenol and copper-zinc-manganese was as efficient as a glucocorticoid-containing cream (triamcinolone acetonide) in reducing adverse reactions after ablative fractional carbon dioxide laser resurfacing used to treat scars of atrophic acne; thus, it may offer an alternative which avoids side effects specific to glucocorticoid application [179].

Lupane-type triterpenes, betulin, betulinic acid, lupeol, lupeolic acid, are the main phytocompounds identified in birch bark extracts (BBE), betulin being the major constituent. A topical oleogel with betulin reached a randomized phase III clinical trial that investigated the re-epithelialization of split-thickness skin graft wounds on 219 patients; results showed the ability of this product to accelerate skin healing and re-epithelialization, thus contributing to burn care practice [180]. The oleogel, Episalvan, was approved by EMA in 2016 for therapeutical use in burn and wound healing and is also studied in preclinical trials as an orphan drug against epidermolysis bullosa [181]. Furthermore, a betulin emulsion showed excellent wound healing features for treating severe necrotizing herpes zoster in a case report presenting a 64-year-old male patient with chronic lymphatic leukaemia and type II diabetes; betulin application provided continuous improving in wound depth, inflammation, pruritus, pain and induced a complete healing without scarring within 8 weeks [182]. Moreover, a semisolid oleogel with a content of 10% triterpenes dry extract as active ingredient was used in a clinical trial on 50 patients whose epidermal recovery and scar aspect in the case of CO2 laser wound were monitored [183]; the active principles are formed from approximatively 80% betulin and 20% other triterpenes (betulinic acid, oleanolic acid, lupeol, erythrodiol). After three months the wounds treated with this oleogel formed a discreet scar in about 30% of patients compared to 10% in the case of standard care. Further monitoring of the scar concluded that the use of the semisolid oleogel improves the chances of a smooth scar formation and thus can be considered for a well-balanced healing.

Among the oleanane-type triterpenes, glycyrrhizin, under the form of licorice extract, an ancient remedy for gastro-duodenal ulcers, was investigated for its wound healing potential in recurrent aphthous stomatitis; a controlled randomized double blind clinical trial evaluated the effect of licorice mouthwash containing diphenhydramine, results indicating a significant reduction in the severity of pain and healing time [184]. Licorice extract and glycyrrhizin in combination with gentian root and willow bark extract were also studied for their implication in atopic dermatitis in a clinical trial that enrolled 10 patients; the general observation was a strong reduction of the inflammatory process in the affected area, comparable with 1% hydrocortisone acetate treatment [185]. In another study, a combination of standardized extracts from *Glycyrrhiza glabra, Thymus vulgaris, Vitis vinifera, Alpinia officinarum* and *Urtica dioica* under the trade name Ankaferd blood stopper was reported to improve wound healing and promote hemostasis in gastro-intestinal bleedings [186]. *Calendula officinalis*, a well-known remedy for wound healing and burns, rich in terpenoids such as oleanane and lupane derivatives, was found to be an effective option in the treatment of diabetic foot ulcers and acute skin damage after cancer radiation therapy by several clinical trials. Lately, the aqueous alcoholic and lipophilic extract of *Calendula officinalis* was approved by EMA for the treatment of minor wounds and inflammations [187].

Lanostane-type triterpenes such as cycloastragenol, the main phytocompound found in Astragalus membranaceus, were found to have implications in wound healing; in a small clinical study, that investigated the physiological immune response after enteral treatment with Astragalus membranaceus extract, results highlighted the immune stimulation and wound healing promotion by changes induced in specific cytokine populations and platelet count [188].

## 6. Conclusions

Wounds and wound healing represents a wide spread health issue, especially if the deeper layers of the skin are affected and/or a chronic lesion is involved. Among the therapeutical strategies and protocols applied in wound healing, plant based remedies play an important role since they were used empirically since ancient times, hence they have stood the test of time. Recently, a high amount of scientific studies were conducted to discover the underlying molecular mechanism of various phytochemicals and plant extracts in order to provide an evidence based validation of their efficacy in wound healing.

This review is focused on pentacyclic and tetracyclic triterpene derivatives with lupane, ursane, oleanane, dammarane, lanostane and cycloartan skeleton, summarizing both their pre-clinical evaluation by in vitro and in vivo assays (Table 1) combined with their clinical assessement. Among these derivatives, the following phytocompounds were outlined: betulinic acid, betulin, lupeol, asiaticoside, asiatic acid, madecassoside, madecassic acid, oleanolic acid, glycyrrhizin, camellioside B, perennisosides, asterbatanoside D, bernardioside B2, bellissaponins, ginsenosides, bacosides, cycloastragenol, astragaloside IV, cyclocephaloside I and cyclocanthoside E. The main biological activities in wound healing, demonstrated in the pre-clinical assays, included accelerated healing process by antioxidative and anti-inflammatory activity, promotion of angiogenesis and type I collagen synthesis, reduction of tissue edema and formation of the skin barrier. Furthermore, some of these phytocompounds induced accelerated wound contraction and closure, shortening of the re-epithelization process and finally, yet importantly, prevention of keloid formation. Many of these biological activities were validated through several clinical trials, which in addition have demonstrated their potential in improving microcirculation and vascular permeability or reducing edema. Moreover, some of these types of triterpenes show potential in dermato-cosmetology due to their ability to reduce stretch marks development, liposclerosis improvement or anti-aging effects. Nonetheless, some of the compounds showed great healing potential in more severe types of wounds, such as diabetic ulcerations, necrotic ulcerative lesions, necrotizing herpes zoster, atopic dermatitis, epidermolysis bullosa or gastro-intestinal bleedings.

To improve the bioavailability, skin permeability and enhance the efficacy of these phytocompounds/plant extracts in wound healing, innovative topical formulations were developed. The first types of formulations included oleogels and hydrogels that were further completed with nanotechnology strategies such as nanoemulsions, nanohydrogels, nanofibers, nanoliposomes or metallic nanoparticles.

Due to their implication and demonstrated pharmacological activities in all stages of wound healing, pentacyclic and tetracyclic triterpenes are a viable option for treating a wide variety of wounds, in several types of formulations that provide both efficacy and safety.

## Figures and Tables

**Table 1:** Triterpenoids with in vitro wound healing effects.

Phytocompound	Source	In vitro Method/Model	Biological Activity	Reference
**Betulinic acid**	*Dillenia indica*	Lipid peroxidation test/egg yolk	Protection against lipid peroxidation	[2]
	*Diospyros kaki*	Lipopolysaccharide-stimulated RAW264.7 macrophages	HO-1/Nrf2 translocation suppressing the NF-κB pathway	[3]
	Pure compound	Rat cardiomyocyte-derived H9c2 cell/hypoxia/reoxygenation (H/R) model	Protection against myocardial ischemia reperfusion injury	[4]
**Betulin**	Birch bark	Human keratinocytes	Keratinocyte migration, increase of pro-inflammatory mediators	[5]
**Lupeol**	*Bowdichia virgilioides* Pure compound	Epidermal keratinocytes and dermal fibroblasts/scratch wound healing assay UVA radiated dermal fibroblast	Enhancement in migration and wound closure and contraction of dermal fibroblasts; regulation via PI3K/Akt and MAPK pathways Anti -aging effects by inhibiting p16, p21 and p-p53 and decreasing the MMP-1, -2, -3 expression.	[6,7]
**Asiaticoside**	Pure compound	Human dermal fibroblasts, human epidermal keratinocytes	increased migration rates of skin cells; enhance the initial skin cell adhesion; increase in the number of normal human dermal fibroblasts	[8]
	Pure compound	Human dermal fibroblasts	Increases the synthesis of type I collagen by activation of the Smad pathway	[9]
	*Centella asiatica*	HaCaT keratinocytes	Pro-migratory effect; upregulation of signaling pathways involved in wound healing:FAK, Akt, and MAPK	[10]
	Pure compound	Keloid primary fibroblast cultures	Inhibition of keloid fibroblasts proliferation and prevention of excessive scarring	[11,12]
**Asiatic acid**	Pure compound	Primary keloid and normal fibroblasts	Keloid prevention by inhibiting TGF-β1-induced collagen expression via PPAR-γ activation	[13]
**Oleanolic acid**	*Viscum album*	NIH/3T3 and HaCat cells/wound healing assay	Enhanced wound closure by stimulation of the migration of fibroblasts	[14]
	Pure compound	mink lung epithelial cells, MDA-MB-231	Stimulation of cell migration by stimulation of mitogen-activated protein (MAP) kinases	[15]
**Glycyrrhizin**	Pure compound	Normal human dermal fibroblasts	Reduction of fibrosis, increase of apoptosis and reduction of autophagy in keloids by HMGB1 inhibition	[16]
**Camellioside B**	*Camellia japonica*	Normal human neonatal skin Fibroblasts	Enhanced proliferation	[17]
**Perennisosides Asterbatanoside D Bernardioside B_2_, Bellissaponins**	*Bellis. perennis*	Normal human dermal fibroblasts	Promotion of collagen synthesis	[18]
**Ginsenosides**	*Panax ginseng* CA Meyer	Human dermal fibroblast cells	Healing effect, increase in type I collagen synthesis by activating the Smad pathway	[19]
	Pure compound	HaCaT/wound scratch assay	Wound healing stimulation by increasing the migration of human keratinocytes through S1P dependent mechanism.	[20]
**Cycloastragenol**	*Astragalus membranaceus*	Human HaCaT keratinocytes and primary human dermal fibroblasts/Scratch wound test	Increase cell migration and proliferation by EGFR stimulation	[21]
	Pure compound	Human epidermal stem cells EpSCs	Wound healing by stimulation of EPSCS proliferation and migration by activation of Wnt/β-catenin signaling	[22]
**Astragaloside IV, Cyclocephaloside I Cyclocanthoside E**	Pure compounds	human keratinocytes/migration scratch assay	Wound healing by proliferation and migration	[23]
**Astragaloside VI**	*Astragalus membranaceus*	Human HaCaT keratinocytes and primary human dermal fibroblasts	Stimulation of skin cell proliferation and migration by activation of EGFR,	[21]

**Table 2:** Triterpenoids with in vivo wound healing effects.

Phytocompound	Source	In Vivo Method/Model	Biological Activity	Reference
***1. EXCISION/INCISION WOUNDS MODEL***				
**Betulin**	Birch bark	Pig ear/porcine ex vivo excision wound healing model and re-epithelialization	Formation of the skin barrier, wound healing, re-epithelization	[5]
**Lupeol**	*Bowdichia virgilioides*	Male Wistar rats/streptozotocin induced hyperglycemia- excision wound model	Enhancement of the healing process through the anti-inflammatory effect of NF-*κ*b signaling pathways	[24]
**Asiaticoside**	*Centella asiatica*	Rabbits/excision wound	Shortening of the epithelization period by increase in hydroxyproline content and induction of collagen synthesis	[25]
	Pure compound	Rabbits/excision wound	Accelerated wound healing, keloid prevention formation, invisible scar formation in open wounds showing tissue loss	[26]
	Pure compound	Guinea pigs/excision wound	Enhanced rate of wound healing by increase in collagen synthesis and tensile strength of the wound tissues	[27]
**Oleanolic acid**	*Anredera diffusa*	Male mice/excision wound	Enhanced cicatrizant activity	[28]
**Bacosides**	*Bacopa monnieri*	Swiss Wistar strain rats/incision wound models	Acceleration of epithelialization and wound contraction rate	[29]
**Astragaloside IV, Cyclocephaloside I Cyclocanthoside E**	Pure compounds	Sprague–Dawley male rats/incision wound model	Wound healing by enhanced cell density, regularly organized dermis and angiogenesis	[23]
**Astragaloside IV**	Pure compound	Sprague-Dawley (SD) female rats/excision model	Faster wound closure by increased collagen synthesis and TGF-β1 levels	[30]
	Pure compound	Sprague–Dawle female rat/skin excision wound model	Acceleration of the wound re-epthelization, angiogenesis, scar prevention	[31]
***2. BURN/THERMAL WOUNDS MODEL***				
**Asiaticoside**	*Centella asiatica*	Male Balb/c mice/burn wound model	Increase in burn wound repair by VEGF and IL-1β production	[32]
	Pure compound	Sprague-Dawley rats/burn wound model	Accelerated skin recovery in deep partial-thickness burn injury by VEGF prodiuction	[33]
**Madecassoside**	*Centella asiatica*	Male ICR mice/burn wound model	Accelerated burn wound healing by increased antioxidative activity, collagen synthesis and angiogenesis.	[34]
	Pure compound	Male Sprague-Dawley rats/burn wound model	Accelerated burn wound healing, wound contraction by stimulation of collagen synthesis, reducing oxidative stress and inducing vasodilatation	[35]
	Pure compound	SD rats/burn wound model	Scar reduction and wound healing improvement	[36]
**Glycyrrhizin**	Pure compound	BALB/c mice/burn wound model	Restores the synthesis of β-defensins and enhances the resistance to infection with *Pseudomonas aeruginosa*	[37]
	Pure compound	Male Sprague-Dawley rats/thermal injury model	Anti-inflammatory effect and organ protection by inhibition of HMGB1	[38]
**Ginsenosides**	*Panax ginseng* CA Meyer	Male Balb/c mice/burn wound model	Reduction of wound area, enhanced wound healing by increased neovascularization and VEGF production	[39]
**Bacosides**	Pure compound	New Zealand Albino rabbits/thermal injury model	Reduction of the scarring area and scarring thickness by downregulation of MMP-1 or TGF-β1 proteins	[40]
***3. SKIN FLAP WOUNDS MODEL***				
**Betulinic acid**	Pure compound	Male C57BL/6 mice/random-pattern skin flap model	Promotion of angiogenesis, reduction of tissue edema, increase in the survivability of the skin flap	[41]
**Asiaticoside**	Pure compound	Male Sprague–Dawley rats/experimental model of rat skin flaps	Enhancement in microcirculation and viability of the skin flaps	[42]
***4. DIABETIC WOUNDS MODEL***				
**Asiaticoside**	Pure compound	SPF SD male rats/diabetic wound model	Accelerated healing of diabetic cutaneous ulcers by regulating Wnt/β-Catenin signaling pathway	[43]
	Pure compound	Sprague Dawley male rats/diabetic wound model	Enhanced rate of wound healing by increase in collagen synthesis and tensile strength of the wound tissues	[27]
***5. GASTRIC ULCER MODEL***				
**Oleanolic acid**	*Fabiana imbricata*	Male Swiss Albino mice/induced gastric ulcer model	Gastroprotective effect	[44]
	*Fabiana imbricata*	Male Sprague–Dawley rats/induced gastric ulcer model	Regeneration of the lesions, increase in gastric mucosal thickness	[45]
***6. INFECTED WOUND MODEL***				
**Cycloastragenol** **(cycloastragenol-6-*O*-beta-D-glucoside)**	*Astragalus membranaceus*	Male C57BL/6JNarl mice/infected wound healing	Wound healing activity by stimulation of angiogenesis	[21]
**Astragaloside VI**	*Astragalus membranaceus*	Male C57BL/6JNarl mice/infected wounds	Promotion of cutaneous wound healing by enhanced angiogenesis	[21]
***7. PSORIASIS MODEL***				
**Betulinic acid**	*Dillenia indica*	Ultraviolet induced psoriasis-like wounds/male albino Wistar rats	Accelerated healing process	[2]
***8. EDEMA MODEL***				
**Asiatic acid**	Pure compound	ICR mice/hind paw edema model	Anti-inflammatory activities, pain relief by inhibition of iNOS, COX-2, interleukin-6, IL-1*β*, and TNF-*α* expression	[46]
***9. AORTIC CONTRACTION/RELAXATION MODEL***				
**Betulinic acid**	Pure compound	Male Sprague Dawley rats exposed to LPS/aortic contraction-realaxation in sepsis	Reduction in impairments of aortic contraction; antiinflamatory effect	[47]

## Data Availability

All data used to support the findings of this study are included within the article.
